# The Role of Intelligence and Self-Concept for Teachers’ Competence

**DOI:** 10.3390/jintelligence10020020

**Published:** 2022-03-28

**Authors:** Sigrid Blömeke, Lars Jenßen, Michael Eid

**Affiliations:** 1 Centre for Educational Measurement (CEMO), University of Oslo, P.O. Box 1161, Blindern, 0318 Oslo, Norway; 2 Department of Primary Mathematics Education, Humboldt-Universität zu Berlin, Unter den Linden 6, 10099 Berlin, Germany; lars.jenssen@hu-berlin.de; 3 Department of Education and Psychology, Freie Universität Berlin, Habelschwerdter Allee 45, 14195 Berlin, Germany; michael.eid@fu-berlin.de

**Keywords:** fluid intelligence, Cattell–Horn–Carroll theory, competence, academic self-concept, mathematics content knowledge, mathematics pedagogical content knowledge, general pedagogical knowledge, early childhood education, early childhood teachers

## Abstract

Research on intelligence and competence has developed widely independent of each other. The present paper aims at relating these traditions and at integrating the dominant models to fill gaps in the respective theories. We test the structural models derived from this integration in a series of confirmatory factor analyses and a latent moderated structural equations approach using teachers as an example. The data reveal that both fluid intelligence (*gf*) and domain-specific knowledge affect teachers’ ability to solve the domain-specific items. Teachers’ academic self-concept related to mathematics explains individual differences beyond *gf*. An interaction effect between *gf* and self-concept exists for teachers’ pedagogical content and general pedagogical knowledge, but not for their mathematics knowledge. This finding indicates that a positive self-concept cannot compensate for a lack of *gf*, but it supports the acquisition of domain-specific knowledge in case of high *gf*, probably because it facilitates overcoming challenges.

## 1. Introduction

The term *intelligence* is used in cognitive psychology to describe a variety of cognitive abilities at different hierarchical levels. More general abilities are assumed to underly more specific ones (Jensen 1998). In educational research in contrast, there are hardly any studies using the term intelligence. Instead, a competing terminology has become prominent that uses the term *competence* as the disposition underlying human behavior (see, e.g., Blömeke et al. 2015).

The aims of the present paper are two-fold: Firstly, we intend to relate the competence terminology prominent in education to the intelligence terminology prominent in cognitive psychology and to integrate the dominant models from these two traditions. Both academic disciplines could benefit from such an integration because it has the potential to fill gaps in the respective theories. Secondly, we intend to test the models we can derive from an integrated theory using teachers as an example.

## 2. Conceptual Framework

### 2.1. The Cattell–Horn–Carroll Theory of Cognitive Abilities

There are still some unresolved incongruencies between different traditions of modeling intelligence. Nevertheless, it is possible to summarize that the Cattell–Horn–Carroll (CHC) theory of cognitive abilities has evolved as a theory widely accepted in cognitive psychology and supported by validity evidence (Flanagan and Harrison 2012; Schneider and McGrew 2012). The CHC theory is based on Carroll’s (1993) *three stratum model* and Cattell’s (1963) and Horn’s (1968) extended *gf*-*gc* model. It describes the hierarchical organization of cognitive abilities where broader mental processes are placed on higher levels and more task-specific abilities on lower levels in the system of human cognitions. Cognitive abilities on higher levels are hypothesized to affect the acquisition of abilities on lower levels. Consequently, individuals with high achievement in one domain often show high achievement in other domains (Gustafsson and Undheim 1996).

There are a variety of cognitive abilities included in the CHC model. From an educational perspective, the most important distinction is between those that are difficult to change because they are, to a large extent, caused by biological and neurological factors and those that are mostly developed through opportunities to learn. *Gf* (fluid intelligence) is the ability to process information and to analytically distinguish between objects. Inductive and deductive reasoning are crucial indicators and regarded as particularly relevant when a situation is new, and automatic processes no longer work (Schneider and McGrew 2012). *Gf* is a dimension commonly regarded as relatively difficult to change, and if so, only over long time (Ritchie and Tucker-Drob 2018). 

In contrast, *gc* (crystallized intelligence) includes a broad class of cognitive abilities mostly acquired through opportunities to learn. A recent development has been to split up this construct into cognitive abilities related to the broader culture and society on the one hand (called *gc*) and domain-specific knowledge (called *gkn*) on the other hand (Schneider and McGrew 2018). The latter dimension is an umbrella term for “specialized knowledge (knowledge not all members of a society are expected to have)” (Schneider and McGrew 2012, p. 123). It is acquired through “intensive systematic practice and training (over an extended period of time)” and is maintained through “regular practice and motivated effort (a.k.a., expertise)” (McGrew 2009, p. 6). *Gkn* includes a range of distinct cognitive abilities (Flanagan and Dixon 2014).

Another CHC dimension that is developed through opportunities to learn, already included in Cattell’s (1963) and Horn’s (1968) extended *gf*-*gc* model, is *gq* (quantitative knowledge). *Gq* describes the “knowledge related to mathematics” (Schneider and McGrew 2012, p. 127).

From an educational perspective, it is crucial to identify cognitive abilities that are mostly developed through opportunities to learn and to distinguish them from those that are more difficult to change. Only then can we design education in line with the effort needed to support human development. Moreover, only then we can evaluate the effects of education properly because the proportion of variance explained by general cognitive ability can be controlled for (McClelland 1973).

Disentangling teacher cognitions in such a way is urgently needed. The sample used in this study was drawn from the population of early childhood teachers because, to our knowledge, there has not yet been a study that has attempted this, even though the value of education has been repeatedly questioned for this group of teachers (Strauss 2005). Without clarifying the role of general cognitive abilities, it is almost impossible to evaluate to what extent such criticism is justified or how to design teacher education so that it supports the development of teacher competence.

### 2.2. Relation of the Competence Model to the CHC Model

In contrast to intelligence models that intend to identify and classify cognitive abilities in a *top-down* manner, competence models intend to identify and classify the dispositions underlying observable behavior. Spencer and Spencer (1993, p. 9) defined competence in this sense as “an underlying characteristic of an individual that is causally related to criterion-referenced effective and/or superior performance in a job or situation. Underlying characteristic means that the competency is a fairly deep and enduring part of a person’s personality.” One could describe this modeling approach as a *bottom-up* approach, since it first identifies the criterion and then tries to identify the traits involved.

The most important goal with modeling competencies in this way is to identify and describe those competence dimensions that are learnable and thus can be influenced by education. Koeppen et al. (2008) defined competence in this sense as “domain-specific cognitive dispositions that are required to successfully cope with certain situations or tasks, and that are acquired by learning processes” (p. 68). Blömeke et al. (2015) extended this understanding of competence as purely cognitive by suggesting considering dispositions as a multi-dimensional set of not only cognitive, but also affective-motivational-volitional characteristics. In their argumentation, they refer back to Snow’s (1994) concept of two pathways that contribute to achievement, namely a cognitive and a commitment pathway, and the broad range of studies that support such a concept. Students’ cognitive abilities are typically the strongest predictors of student achievement. However, including motivational and similar characteristics increases the predictive validity, although to a smaller extent, particularly with respect to teacher-set grades, but less so with respect to standardized test scores (Lavrijsen et al. 2021; Steinmayr et al. 2019). Academic self-concept proved to be the most influential construct in this context (ibid.).

The Blömeke et al. (2015) competence model further clarified that these cognitive and affective-motivational dispositions are domain-specific, but in a general way, beyond single tasks and situations within this domain. The dispositions are stable continuous traits that, in turn, underlie task- and situation-specific cognitive skills not organized in an academic-disciplinary way, as is knowledge, but along the specific demands of narrowly defined situations or tasks. Blömeke et al. (2015) propose regarding the transformation of dispositions into observable behavior as fully or partially mediated by task- and situation-specific cognitive skills.

From this description, it should be clear that there is strong overlap between the CHC and the Blömeke, Gustafsson, and Shavelson competence models. The cognitive dimension of competence is conceptualized similarly to *gkn* as domain-specific and learnable. It is highly specialized knowledge that has been acquired during a long process of education and requires regular practice. We are not the first ones to point out such an overlap of competence and intelligence models. Previously, Wilhelm and Nickolaus (2013) and Trapp et al. (2019) had done this, although they did not specify the overlap in detail. 

The affective-motivational-volitional dimension of the Blömeke, Gustafsson, and Shavelson competence model overlaps with the investment traits as conceptualized by Ackerman (1996) and Ziegler et al. (2012). The investment theory has provided evidence for the existence of traits that support the development of certain cognitive abilities because these support “the tendency to seek out, engage in, enjoy and continuously pursue opportunities for effortful cognitive activities” (von Stumm et al. 2011, p. 225). Ackerman (1996) examined a set of personality and motivational traits and provided evidence for their effects on *gc* beyond the effects of *gf*. Ziegler et al. (2012) extended this research with the openness-fluid-crystallized-intelligence model by providing evidence for an interaction effect of *gf* and personality traits, in particular, openness. However, very few studies exist that test the interaction hypothesis (e.g., (Zhang and Ziegler 2015) with respect to the interaction of openness and *gf*; (Lechner et al. 2019) with respect to the interaction of openness, domain-specific interest, and *gf*).

The core differences between the intelligence and competence traditions are, first, the absence of general cognitive abilities that are difficult to change, in particular of *gf*, in the Blömeke, Gustafsson, and Shavelson model. Although this is a decision that was made intentionally, since the objective was to identify traits that can be influenced by education, this absence could play out in an unintended negative way. An evaluation of the effects of education could be hampered by hidden third-variable (e.g., *gf*) effects. Conceptually, it can be argued that *gf* is less relevant for experts because their specialized knowledge can only be gained through specific opportunities to learn during many years of education. However, specialized knowledge also represents cognitive traits where investments should be crucial, since we know from previous research that *gf* shows effects in all cognitive domains. Many studies and meta-analyses demonstrate, for example, that *gf* is able to explain substantial amounts of variance in domain-specific school achievement (e.g., Brunner 2008; Gustafsson 1994).

Second another core difference between the two traditions is the role of the criterion. Both the CHC and the competence models are structural theories that examine the dimensions and facets of the respective constructs. While no criterion per se is considered in the intelligence tradition, modeling competence first identifies the criterion in terms of human behavior and then examines the underlying dispositions. 

Third, the investment theory has so far focused on selected personality and motivational traits. Self-related cognitions have not been included, even though they would be considered a dimension of competence models given that studies on predictors of student achievement point to the relevance of self-related cognitions when it comes to explaining individual differences (e.g., Marsh and Martin 2011). Most recently, Demetriou et al. (2019) examined relationships between school achievement, *gf*, and self-concept using a sample of 10- to 17-years-olds. They found a decreasing effect of *gf* on school achievement but an increasing effect of self-concept with age. Since we intend to examine the cognitive structure of teachers, this result is very relevant. 

### 2.3. Teachers’ Competencies

Teachers acquire their competencies through a long process of schooling, teacher education, and professional development. This also applies to early childhood (EC) teachers used as a sample in this study (Dunekacke et al. 2021). Based on the seminal work by Shulman (1987), teachers’ professional knowledge can be modelled as a three-dimensional construct that includes content knowledge, pedagogical content knowledge, and general pedagogical knowledge. With respect to content, the present study focuses on the domain of mathematics and EC teachers’ task to support children’s mathematical learning in early childhood education. Their professional knowledge then includes mathematics content knowledge (MCK), mathematics pedagogical content knowledge (MPCK), and general pedagogical knowledge (GPK) (Dunekacke et al. 2021). 

MCK is the knowledge about numbers, sets, and operations; shape, space, and change; quantity, measurement, and relations; data, combinatorics, and chance (Bruns et al. 2021; Dunekacke et al. 2015a). In the case of EC teachers, this dimension has strong conceptual overlap with *gq*, since the level of mathematics does not exceed the level of mathematics learned during schooling. There are rarely opportunities to learn mathematics in EC teacher education that go beyond this level of mathematics. However, note that in case of other teacher groups, for example those trained for teaching mathematics in upper-secondary schools, MCK acquired during teacher education would include elements of university mathematics. This type of MCK would have to be classified as *gkn* because it is highly specialized mathematical knowledge which we cannot expect that all or even many members of a society have.

The MPCK of EC teachers is the knowledge of how to diagnose children’s developmental state in mathematics and how to design an informal learning environment that supports the mathematical learning of children between age 3 and 6 (Dunekacke et al. 2015b; Lee 2010; Torbeyns et al. 2020). GPK includes general foundations from educational theory, psychology, and instructional research related to early childhood and learning processes of 3- to 6-year-olds (Blömeke et al. 2017; Malva et al. 2019). 

This framework, the alignment of the framework and its measures, as well as the inferences to be drawn from these measures have been validated in a range of studies (Blömeke et al. 2017; Dunekacke et al. 2015a, 2015b; Jenßen et al. 2015a, 2015b; Blömeke et al. 2022). The data for these studies were collected in Germany. However, the framework also reflects the professional tasks and opportunities to learn of EC teachers in other countries (e.g., Clements et al. 2004; NAEYC 2009).

As both the Blömeke, Gustafsson, and Shavelson model and the investment theory indicate, a purely knowledge-based approach to explaining individual differences in EC teachers’ *gq* (MCK) and *gkn* (MPCK and GPK) may be limited given the long educational process underlying the development of these knowledge dimensions. Academic self-concept can be defined as an individual’s belief about their abilities. Such self-related cognitions have been found to be relevant for a broad range of developmental and educational outcomes (Marsh et al. 2016). According to Marsh (1990) and Bong and Skaalvik (2003), academic self-concept is a domain-specific construct and needs to be examined with scales specific for the cognitive construct under investigation. Modeling the dispositions underlying human behavior in such a way may represent a more holistic perspective, thus enabling the explanation of more variance. 

### 2.4. The Structure of EC Teachers’ Competencies

Based on the theory described above, *gf*, *gq*/MCK, and *gkn* (MPCK and GPK), as well as academic self-concept, are core dispositions involved in EC teachers’ work of supporting children’s mathematical development. However, there is ambiguity with respect to their structure and hierarchy. Carroll (1993) had placed more specialized cognitive abilities on the third, or lowest, level of his taxonomy. In contrast, the most recent CHC theory places *gf*, *gq*, and *gkn* on the second level and narrower abilities on the level below (Flanagan and Dixon 2014). 

Evidence for the relation of *gf* to the other constructs has been provided by comparing first-order models with hierarchical models. The latter typically fit better to the data (Demetriou et al. 2019). In one-dimensional first order models, *g* explains variance in all indicators without distinguishing between the different constructs. In correlated first-order models, specific constructs are distinguished but still placed on the same level. In hierarchical models, *gf* also influences the specific constructs and thus, indirectly, their indicators (Gignac 2008). In this case, the effects of *gf* are fully mediated by the specific factors (Yung et al. 1999). Alternatively, *gf* directly influences the indicators of the different specific constructs as part of a hierarchical bifactor (Holzinger and Swineford 1937) or nested-factor model (Gustafsson and Balke 1993).

The exact role of academic self-concept is also an open question. In an early study, Gose et al. (1980) provided evidence for the additional potential of self-concept to explain variance in reading, language, and mathematics student achievement beyond *gf*. Later on, a study by Schicke and Fagan (1994) supported this result with respect to several student cohorts at different grade levels. Further studies revealed similarly positive relations of self-concept to domain-specific achievement beyond the effects of *gf* (Lavrijsen et al. 2021; Steinmayr et al. 2019). In addition, Guo et al. (2016) pointed to a potential interaction of self-concept and *gf*.

Not surprisingly, given the absence of *gf* in competence models and corresponding research, the organization of EC teachers’ domain-specific cognitions and their relation to broader general cognitions can be regarded as a desideratum. An additional reason for this desideratum might be that standardized and validated assessments covering EC teachers’ professional knowledge are rare (European Commission et al. 2019). Therefore, as yet we have only limited research with respect to the structure of EC teachers’ competencies, restricted to a few knowledge dimensions and to preservice EC teachers still in teacher education. The same restrictions apply to other teacher groups (Kleickmann et al. 2013 examined preservice primary and secondary school teachers; Roloff et al. 2020 studied practicing secondary school teachers).

With respect to preservice EC teachers (*n* = 353), a study by Jenßen et al. (2019) revealed a strong impact of *gf* (in terms of verbal, numerical, and figural intelligence assessed with the screening version of I-S-T 2000R; Liepmann et al. 2012) on MCK and MPCK. Nevertheless, a nested model revealed that MCK and MPCK had a significant additional impact on the ability to solve the domain-specific items. The initially strong latent correlation between MCK and MPCK, if *gf* was not controlled for (*r* = .67), could largely be explained by *gf* modelled as a higher-order factor. The size of the correlation was reduced substantially in the nested model (*r* = .30). Moreover, as typical for more complex models, the nested-factor model fit better to the data than a pure *g*-factor model where all indicators loaded on one general factor *g* only. The nested model also explained more variance.

Blömeke and Jenßen (2016) used data from the same sample to examine the impact of *gf* on the relationship between preservice EC teachers’ MCK or MPCK, respectively, and their skill for perceiving specific mathematics-related situations. A latent regression of this perception skill on the two knowledge constructs without including *gf* revealed strong effects (MPCK: *β* = .64, MCK: *β* = .47). The decrease in the effects of MCK or MPCK, respectively, on the skill to perceive mathematics-related situations (MPCK: *β* = .42, MCK: *β* = −.08) in a nested model including *gf* revealed the relevance of the latter for the interplay between the constructs, particularly with respect to the relationship between MCK and the skill to perceive math-related situations. The nested models were additionally tested against second-order models. Differences in the results were negligible.

We have not found any study that examined the cognitive structure of practicing teachers or that included GPK. Moreover, potential investment traits, such as academic self-concept, have not yet been examined. Our study adds to the body of research in both respects.

### 2.5. Study Aims and Hypotheses

The first aim of the empirical portion of this paper is to clarify the role of *gf* for practicing EC teachers’ domain-specific knowledge. We test the common models against each other with the following hypotheses:

**H1.**  *There are strong positive latent correlations between MCK, MPCK, and GPK if gf is not controlled for.*

**Figure 1:**
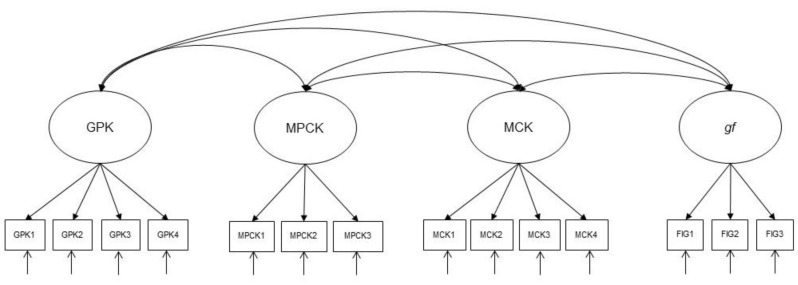
The latent correlation model (H2). Note. MCK = mathematics content knowledge; MPCK = mathematics pedagogical content knowledge; GPK = general pedagogical knowledge; *gf* = fluid intelligence.

**H2.**  *Gf is positively correlated with MCK, MPCK, and GPK (see Figure 1).*

**Figure 2:**
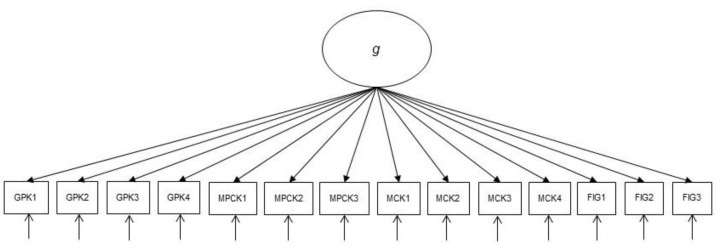
The pure *g* model (H3). Note. MCK = mathematics content knowledge; MPCK = mathematics pedagogical content knowledge; GPK = general pedagogical knowledge; *gf* = fluid intelligence.

**H3.**  *A pure g model, where all indicators load on one factor (see Figure 2), reveals an inacceptable model fit because it does not take into account the differential effects of gf, MCK, MPCK, and GPK on EC teachers’ ability to solve the corresponding items.*

**Figure 3:**
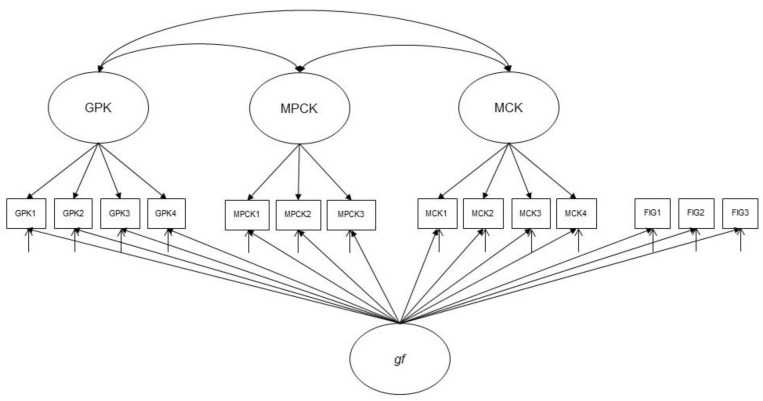
The bifactor (S-1) model (H4). Note. MCK = mathematics content knowledge; MPCK = mathematics pedagogical content knowledge; GPK = general pedagogical knowledge; *gf* = fluid intelligence.

**H4.**  *Gf explains variance in MCK, MPCK, and GPK, but there are positive (partial) correlations between MCK, MPCK, and GPK after controlling for gf in a bifactor (S-1) model (see Figure 3).*

The second aim of the empirical portion of this paper is to clarify the role of academic self-concept for EC teachers’ ability to solve the domain-specific items:

**H5.**  *We hypothesize that EC teachers’ self-concept related to mathematics explains individual differences in teachers’ MCK, MPCK, and GPK beyond gf.*

**Figure 4:**
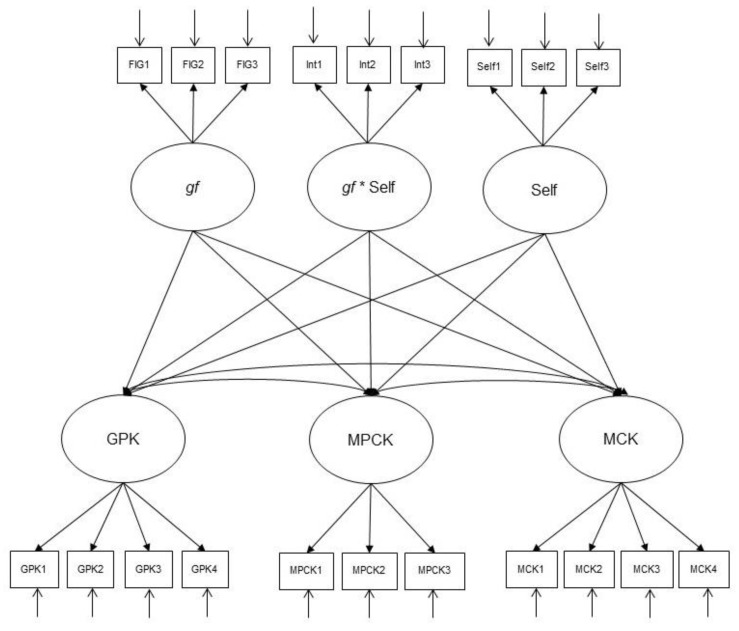
The moderation model (H6). Note. MCK = mathematics content knowledge; MPCK = mathematics pedagogical content knowledge; GPK = general pedagogical knowledge; *gf* = fluid intelligence; Self = self-concept in mathematics; * = interaction.

**H6.**  *Academic self-concept moderates the influence of gf on MCK, MPCK, and GPK (see Figure 4).*

## 3. Materials and Methods

### 3.1. Sample

Practicing EC teachers were the target population of the present study. 210 EC teachers took the test battery. They were recruited by contacting EC institutions from all municipalities in two German federal states (Berlin and Brandenburg) via email and asking for voluntary participation. Therefore, they do not constitute representative samples. EC teachers were surveyed online without any time limitation. If they desired, they could postpone the second part of the survey until the next day. The teachers were, on average, *M* = 28 years old (*SD* = 7.4; min–max = 21–57). The majority (84%) indicated *female* as gender, while 16% indicated *male* as gender. Almost all EC teachers in the sample (93%) spoke German as their first language. About one-third of the sample (34%) had been trained at EC colleges, while the other two-thirds had received their training at post-secondary schools. Their median grade in their last class of school mathematics (a variety of levels, depending on the school year and type of class) was a 3 on a scale from 1 (best) to 6 (worst), with 4 as the minimum passing grade. These characteristics satisfactorily reflect the heterogeneity of EC teachers in Germany. 

### 3.2. Measures of EC Teachers’ Competencies

Similar to Zhang and Ziegler (2015), we used figural reasoning as an indicator of *gf*. This was assessed with the figural facet of the intelligence structure test on the basis of the *Berlin Model of Intelligence Structure (BIS)* (Jäger 1982). Validation studies revealed that the figural subtest of the Intelligence-Structure Test 2000R (Amthauer et al. 2001) provides test scores corresponding to visual-based thinking (Beauducel et al. 2001; Ziegler et al. 2012). The figural facet of the screening version (IST-Screening; Liepmann et al. 2012) consists of 20 items. A CFA applying the WLSMV estimator for categorical data revealed a good fit to the data and supported our one-dimensional assumption (χ^2^ = 191.0, *df* = 170, *p* = .13; RMSEA = .03, 90%CI[.00; .05]; CFI = .94). Scale reliability as indicated by McDonald’s omega for congeneric scales (McDonald 1999) was good (ω = .90, SE = .02). We could therefore build item parcels using a factorial approach by sequentially assigning items to three parcels based on factor loadings, starting with the highest loading. This procedure led to a perfectly identified congeneric measurement model with standardized factor loadings between .64 and .78, applying the MLR estimator. 

With respect to each dimension of EC teachers’ professional knowledge, large item pools were developed in collaboration with academic and practical experts. Item selection was completed on the basis of both conceptual considerations (American Educational Research Association et al. 2014) and a series of cognitive labs, unstandardized pre-pilot, standardized pilot, and validation studies (Jenßen et al. 2015a, 2015b; Dunekacke et al. 2015a, 2015b). The resulting tests consisted of multiple-choice, bundled, and open-response items. In all cases, gender-neutral language was used to reduce the risk of stereotype threats (Cadinu et al. 2005). For item examples, see Appendix A.

MCK was assessed with 23 items covering the four subdimensions described above. McDonald’s omega was very good (ω = .94, SE = .01). A one-factor congeneric measurement showed a good approximative fit (χ^2^ = 377.5, *df* = 230, *p* < .001; RMSEA = .06, 90%CI[.05; .06]; CFI = .99, applying the WLSMV estimator), indicating that the 23 items are approximately unidimensional. Four parcels were built using a substantive approach by assigning five or six items, respectively, to parcels based on the subdimensions leading the test development. A one-factor congeneric measurement model showed a very good model fit (MLR estimator; χ^2^ = 0.4, *df* = 2, *p* = .82; RMSEA = .00, 90%CI[.00; .08]; CFI = 1.00). The standardized factor loadings were between .85 and .94. 

The second facet was mathematics pedagogical content knowledge (MPCK). It was assessed with 35 items. McDonald’s omega was very good (ω = .95, SE = .01). A one-factor congeneric measurement model showed a good approximate fit (WLSMV estimator; χ^2^ = 620.1, *df* = 561, *p* < .05; RMSEA = .02, 90%CI[.01; .03]; CFI = .99). We built three parcels by assigning items to parcels based on their loadings. The parcels showed strong standardized loadings between .92 and .95 in a one-factor congeneric measurement model.

The third facet was general pedagogical knowledge (GPK). It was assessed with 30 items covering four subdimensions. McDonald’s omega was very good (ω = .95, SE = .01). A one-factor congeneric measurement model with all 30 items as observed variables showed a good approximate fit (WLSMV estimator; χ^2^ = 499.3, *df* = 405, *p* < .001; RMSEA = .03, 90%CI[.02; .04]; CFI = .99). We built four parcels based on a substantive approach by assigning items to parcels based on the subdimensions leading test development. A one-factor congeneric model with the four parcels as indicators showed a very good fit to the data (χ^2^ = 0.9, *df* = 2, *p* = .64; RMSEA = .00, 90%CI[.00; .11]; CFI = 1.00) with standardized factor loadings between .82 and .93. 

EC teachers’ academic self-concept was assessed with three domain-specific items related to mathematics. The statements were formulated negatively to maximize variance. We recoded the negatively worded statements to positive ones to facilitate the interpretation. McDonald’s omega was good (ω = .90, SE = .02). A one-factor congeneric measurement model revealed standardized factor loadings between .82 and .93. The three items are (our translation): “Nobody is capable of everything. I just do not have any talent for math;” “Mathematics does not particularly suit me;” “When it comes to things in mathematics that I do not understand, I know from the start: I will never understand this.”

See Table 1 for correlations of all parcels.

### 3.3. Data Analysis

The hypotheses H1 and H2 were analyzed using a CFA with correlated first-order factors for each construct (e.g., Eid et al. 2017b). Hypothesis H3 was tested assuming a one-factor-congeneric measurement model for all observed variables. Hypothesis H4 was analyzed using a bifactor (S-1) model (Eid et al. 2017b; Eid et al. 2018) with the *gf* factor as a general reference factor and three correlated specific factors for MCK, MPCK, and GPK.

Hypothesis H5 was analyzed using a multivariate latent regression analysis with the factors of MCK, MPCK, and GPK as dependent variables and the factors of *gf* and self-concept as independent variables. In order to analyze hypothesis H6, this multivariate regression model was extended by including interactions between *gf* and self-concept in a latent moderated structural equations approach (LMS; Klein and Moosbrugger 2000). Moreover, in this case, a maximum likelihood estimator with robust standard errors was used by applying a numerical integration algorithm. As suggested in the literature, we included the main effects of all variables to avoid the confounding of the main and interaction effects or the possibility that changes in the scale could result in arbitrary estimates (Aiken and West 1991). 

All analyses were carried out with the Mplus 8.4 software package (Muthén and Muthén 1998–2019) using the robust maximum likelihood estimator (MLR). Missing data existed on two constructs, *gf* and academic self-concept, because teachers had dropped out after the knowledge tests. The missingness was handled by using the full-information maximum likelihood (FIML) procedure implemented in Mplus. The FIML procedure provides unbiased parameter estimates and standard errors under the missing-at-random assumption (Enders and Bandalos 2001). Testing the missing-completely-at-random assumption with the background information available revealed that most variables did not display significant differences for teachers with full as compared to those with missing data. This applied to teachers’ age, the level of their school degree, and their cultural capital, as well as to the education background of their fathers and mothers. However, significant differences existed with respect to gender (Cohen’s *d* = −.20, CI = −.59–.19) in favor of female teachers showing fewer missing values and language background (Cohen’s *d* = .42, CI = .02–.81) in favor of teachers with German as the language spoken at home showing fewer missing values.

## 4. Results

### 4.1. Role of EC Teachers’ gf for Their Domain-Specific Knowledge

#### 4.1.1. H1 and H2: Latent Correlations of MCK, MPCK, and GPK, as Well as *gf*

A CFA model with correlated first-order factors for *gf*, MCK, MPCK, and GPK showed a very good fit (χ^2^ = 79.2, *df* = 71, *p* = .24; RMSEA = .02, 90%CI[.00;.05]; CFI = 1.00). The model revealed strong latent correlations between the three dimensions of EC teachers’ knowledge (see Table 2) if *gf* is not controlled for. In particular, MPCK and GPK were empirically almost indistinguishable (*r* = .95). The two domains related to mathematics were less strongly, but still substantially correlated with each other (MCK/MPCK: *r* = .54; MCK/ GPK: *r* = .48). There were also high latent correlations between *gf* and the three knowledge dimensions. The correlation was particularly high for MCK (*r* = .84), and lower, but equally high for MPCK and GPK (*r* = .52). Hence, our analyses support hypothesis H1 and H2.^1^

#### 4.1.2. H3: One-Dimensional Structure of EC Teachers’ Cognitive Abilities

In accordance with our hypothesis (H3), the data revealed an unacceptable model fit for the pure *g*-factor model where just one factor was hypothesized to underly all 14 observed variables, i.e., the indicators of *gf,* MCK, MPCK, GPK (χ^2^ = 898.5, *df* = 77, *p* < .001; RMSEA = .23, 90%CI[.21; .24]; CFI = .67). The standardized factor loadings varied between .37 for two of the *gf* parcels and >.90 for the three MPCK parcels. For the *gf* parcels, the variance explained by the underlying factor was very low (<0.2) and barely significant. The modification indices suggest many correlated error variables, in particular of indicators belonging to the same construct, showing that the structure is multidimensional. Regarding EC teachers’ cognitive abilities as one-dimensional is thus not supported by the data, which is in line with the well-fitting model with correlated first-order factors presented in Table 1, and our hypothesis H3.

#### 4.1.3. H4: Hierarchical Structure of EC Teachers’ Cognitive Abilities: Bifactor (S-1) Model

The bifactor (S-1) model fit the data very well (χ^2^ = 70.3, *df* = 63, *p* = .25; RMSEA = .02, 90%CI[.00; .05] CFI = 1.00). The percentage of variance explained by the *gf* factor was very high for the indicators of MCK (between 54 and 64 percent), and lower, but still substantive, for the indicators of MPCK (between 20 and 24 percent) and PCK (between 13 and 21 percent). The correlations between the specific factors are partial correlations (after controlling for *gf*). These correlations (see Table 3) are smaller than the zero-order correlations presented in Table 2, and not statistically significant for MCK and GPK. Therefore, as a whole, H4 has to be rejected. However, it is noteworthy that the correlation between the two pedagogical knowledge dimensions is still very high. These results show that the general *gf* factor can explain, to a relatively large degree, the correlations between the content knowledge (MCK) and the pedagogical knowledge (MPCK, GPK), but not the correlations between the pedagogical knowledge dimensions.

### 4.2. Role of Academic Self-Concept

The second aim of the empirical portion of this paper was to examine the role of self-related cognitions for the ability to solve domain-specific items. In the first step, we extended the CFA model with correlated first-order variables presented in Table 2 by including a factor for self-concept. This model fit the data approximately well (χ^2^ = 145.3, *df* = 109, *p* < .05; RMSEA = .04, 90%CI[.02; .06]; CFI = .99). Self-concept is significantly correlated with all other constructs (see Table 4). Whereas there were only comparatively small correlations with MPCK and GPK, the correlations were stronger with respect to MCK and *gf*.

The multivariate latent regression model fit the data well (χ^2^ = 145.3, *df* = 109, *p* < .05; RMSEA = .04, 90%CI[.02; .06]; CFI = .99). In line with our hypothesis (H5), EC teachers’ academic self-concept explained individual differences in teachers’ MCK beyond *gf* (see Table 5). However, in contrast to our hypothesis, the regression coefficient was not significantly different from 0 for MPCK and GPK. Both independent variables together explained a substantive amount of variance in all dependent variables, but the variance explained in the dependent variables differed by construct: while the variance in MCK could, to a large extent, be explained by *gf* and self-concept (*R*^2^ = .76), the proportion was lower in the case of MPCK and GPK (*R*^2^ = .27 in both cases).

A latent moderated modeling approach including an interaction effect between *gf* and academic self-concept (see Figure 4) revealed no moderator effect with respect to MCK (see Table 6). This means that H6 must be rejected with respect to this dimension. In contrast, the interaction effect was significant for both MPCK and GPK. This result suggests that in the case of high *gf*, the effect of a positive self-concept is stronger than in the case of low *gf*.

In line with these results, the variance explained in MCK was not higher in the latent moderated approach than in the model without an interaction term, while it was substantially higher in the case of MPCK (*R*^2^ = .36) and GPK (*R*^2^ = .34).

## 5. Discussion

Confronting theories stemming from cognitive psychology and education may benefit both traditions. The CHC theory uses an established terminology and provides a broad picture of how to think about the structure of human cognitions (Schneider and McGrew 2012). A comparison with the Blömeke, Gustafsson, and Shavelson model of competence (2015) revealed that substantial conceptual overlap exists between *gq* and MCK on the one hand and *gkn* and MPCK and GPK on the other hand. However, from an educational point of view, the CHC theory neglects, to some extent, the final objective: explaining human behavior and identifying ways to influence it. The Blömeke, Gustafsson, and Shavelson model of competence, in contrast, emphasizes behavior as the criterion and relates the dispositions needed to show this behavior to the criterion. However, this model does not include broader general cognitive abilities, such as *gf*, beyond the domain-specific ones.

The latter is a challenge not many educators may have yet realized. Relations between achievement domains are at risk for appearing as excessively strong if a common underlying factor is neglected. The effects of opportunities to learn can hardly be evaluated properly if general cognitive abilities are not partialled out. In fact, our study revealed interesting insights with respect to the role of *gf* for the population of EC teachers. A bifactor (S-1) model fitted the data well, which means that both general cognitive ability and domain-specific knowledge influenced EC teachers’ ability to solve the domain-specific items. Competence models are therefore advised to include *gf*—not as a cognitive ability that can be influenced by teacher education to a larger extent, but as a necessary precondition to be considered when evaluating the effects of education.

The benefits but also the challenges of teacher education or professional development are otherwise at risk of remaining hidden, as the following example may reveal: to be able to use their knowledge in practice requires that the different dimensions of EC teachers’ professional knowledge be deeply integrated (Tirosh et al. 2011). In particular, the integration of MCK with the two pedagogical dimensions has been regarded as critical. At first glance, EC teacher education seems to succeed in this respect, given latent correlations around .50 both for MCK and MCPK and for MCK and GPK. However, it now turns out—as we have demonstrated in this article—that these relations were, to a large extent, caused by an underlying factor (*gf*), reducing estimates to around .20 once *gf* was controlled for. Thus, the initial correlations did not reflect integration. It then seems to be important to develop educational practices that have the potential to overcome the disintegration of MCK, MPCK, and GPK, in particular, since we know from previous research that a similar phenomenon of seemingly strong latent correlations also applies to pre-service EC teachers (Jenßen et al. 2019). The latent correlation between MCK and MPCK of this target population was at .67, if *gf* was not controlled for, but only at .30 otherwise.

The investment theory could also benefit from being confronted with models of competence. Defining a criterion and trying to identify underlying traits that support its development may expand the range of characteristics examined. Our data revealed, for example, that academic self-concept affects professional knowledge and can thus be regarded as an investment trait. An interesting, and in case of GPK, even surprising finding was that the interaction effect of *gf* and academic self-concept was significant for both MPCK and GPK, given that self-concept was domain-specifically operationalized. This finding indicates that a positive self-concept cannot compensate for a lack of *gf*, but that it contributes to gaining MPCK and GPK in the case of high *gf*. The underlying mechanism here is probably related to the general finding that a positive self-concept makes it easier to overcome challenges. Moreover, the result may indicate that domain-specific self-concept is related to a broader, more general type of academic self-concept, as pointed out by Marsh (1990).

## 6. Limitations

Our study was the first one that examined the cognitive structure of practicing EC teachers. It was also the first one that included academic self-concept as a potential investment trait and examined its interaction with *gf*. However, given that research on the relation of intelligence and competence is scarce and we only used one sample of EC teachers in Germany, it would be important to replicate our findings with other teacher samples, in particular from other countries and other domains (e.g., oral language or science) to avoid making too far-reaching conclusions. Mathematics is only one domain among others where teachers are expected to support children’s development. Relationships between competence and intelligence, as well as relationships between the different knowledge dimensions, could be different in the case of other domains or other groups of teachers, for example, those teaching in upper-secondary school. Testing whether our findings reflect domain- or age-specific particularities would provide a deeper understanding of the nature of teachers’ cognitive abilities. The same applies to testing the generalizability of our models across countries. Such studies should then also examine whether and which abilities are able to predict the intended long-term outcomes, such as child development.

Another limitation of our study was the operationalization of *gf* as figural reasoning. Although this has been done previously (Zhang and Ziegler 2015), a risk exists that such a narrow representation underestimates relations to constructs based on verbal stimuli (Beauducel et al. 2007) such as GPK. The bifactor (S-1) model revealed in the current operationalization a strong effect of *gf* on GPK. We cannot rule out the possibility that the effect would have been even stronger in case of a broader operationalization that included verbal intelligence. Since it was not possible for us, due to limited administration time, to include such a facet of intelligence, we cannot test this with our current dataset, and we encourage further studies on this relationship.

## 7. Conclusions

Beyond EC teachers, the cognitive structure of other teacher populations such as primary, lower- or upper-secondary teachers is largely an open question. Can we reasonably assume that their cognitive structure is similar to that of EC teachers? Differences in opportunities to learn are huge, starting from different entry requirements to different length and depth of domain-specific teacher education, with EC teachers having by far the lowest number of opportunities to learn, both during teacher education (Gasteiger et al. 2020) and professional development (Nasiopoulou et al. 2021). In that sense, one could even speculate that *gf* might be more relevant in case of EC teachers than of others—but this is an empirical question.

## Figures and Tables

**Table 1:** Correlations between all parcels.

	MCK1	MCK2	MCK3	MCK4	MPCK1	MPCK2	MPCK3	GPK1	GPK2	GPK3	GPK4	IST1	IST2	IST3
MCK2	.80													
MCK3	.87	.79												
MCK4	.87	.78	.85											
MPCK1	.49	.42	.48	.46										
MPCK2	.48	.42	.48	.48	.89									
MPCK3	.48	.42	.48	.45	.88	.86								
GPK1	.42	.34	.44	.43	.81	.82	.82							
GPK2	.36	.32	.34	.35	.76	.75	.73	.77						
GPK3	.36	.30	.37	.38	.74	.78	.78	.80	.69					
GPK4	.42	.39	.44	.41	.77	.81	.77	.80	.70	.75				
IST1	.61	.53	.56	.56	.40	.37	.32	.36	.33	.34	.37			
IST2	.72	.65	.65	.65	.42	.40	.36	.40	.32	.29	.39	.68		
IST3	.65	.60	.65	.60	.40	.38	.33	.38	.27	.26	.37	.60	.65	
Self1	.56	.54	.59	.53	.28	.25	.29	.27	.21	.20	.21	.35	.49	.34
Self2	.50	.46	.54	.47	.22	.21	.24	.16	.20	.12	.16	.34	.51	.28
Self3	.58	.53	.60	.53	.25	.19	.23	.22	.16	.11	.21	.40	.48	.40

MCK = mathematics content knowledge; MPCK = mathematics pedagogical content knowledge; GPK = general pedagogical knowledge; IST = fluid intelligence; Self = academic self-concept.

**Table 2:** CFA model with correlated first-order factor: latent correlations and standard errors (in parentheses).

Construct	MCK	MPCK	GPK
MPCK	.54 (.06) ***		
GPK	.48 (.06) ***	.95 (.02) ***	
*Gf*	.84 (.06) ***	.52 (.08) ***	.52 (.08) ***

MCK = mathematics content knowledge; MPCK = mathematics pedagogical content knowledge; GPK = general pedagogical knowledge; *gf* = fluid intelligence. ***: *p* < .001.

**Table 3:** Bifactor model: Latent partial correlations between the specific factors (standard errors in parentheses).

MCK–MPCK	MPCK–GPK	MCK–GPK
.26 (.12) *	.94 (.02) ***	.18 (.12)

MCK = mathematics content knowledge; MPCK = mathematics pedagogical content knowledge; GPK = general pedagogical knowledge; *gf* = fluid intelligence. *: *p* < .05, ***: *p* < .001.

**Table 4:** CFA model with correlated first-order factor: latent correlations and standard errors (in parentheses).

	MCK	MPCK	GPK	*gf*
MPCK	.54 (.06) ***			
GPK	.48 (.06) ***	.95 (.02) ***		
*gf*	.84 (.06) ***	.52 (.08) ***	.52 (.08) ***	
Self-concept	.66 (.08) ***	.30 (.09) **	.26 (.09) **	.57 (.09) ***

MCK = mathematics content knowledge; MPCK = mathematics pedagogical content knowledge; GPK = general pedagogical knowledge; *gf* = fluid intelligence. **: *p* < .01, ***: *p* < .001.

**Table 5:** Latent multivariate regression model: standardized regression coefficients for the effects of the independent variables *gf* and academic self-concept on MCK, MPCK, and GPK (standard errors in parentheses).

	MCK	MPCK	GPK
*Gf*	.69 (.10) ***	.52 (.12) ***	.54 (.12) ***
Self-concept	.28 (.11) *	.01 (.12)	−.05 (.12)

MCK = mathematics content knowledge; MPCK = mathematics pedagogical content knowledge; GPK = general pedagogical knowledge; *gf* = fluid intelligence. *: *p* < .05, ***: *p* < .001.

**Table 6:** Latent moderated structural equation model: standardized regression coefficients for the effects of the independent variables *gf* and academic self-concept, and their interaction on MCK, MPCK, and GPK (standard errors in parentheses).

	MCK	MPCK	GPK
*Gf*	.73 (.12) ***	.55 (.14) ***	.58 (.13) ***
Self-concept	.21 (.13)	−.14 (.14)	−.17 (.13)
*gf* x self	.03 (.06)	.30 (.07) ***	.24 (.06) ***

MCK = mathematics content knowledge; MPCK = mathematics pedagogical content knowledge; GPK = general pedagogical knowledge; *gf* = fluid intelligence; self = self-concept. ***: *p* < .001.

**Figure A1:**
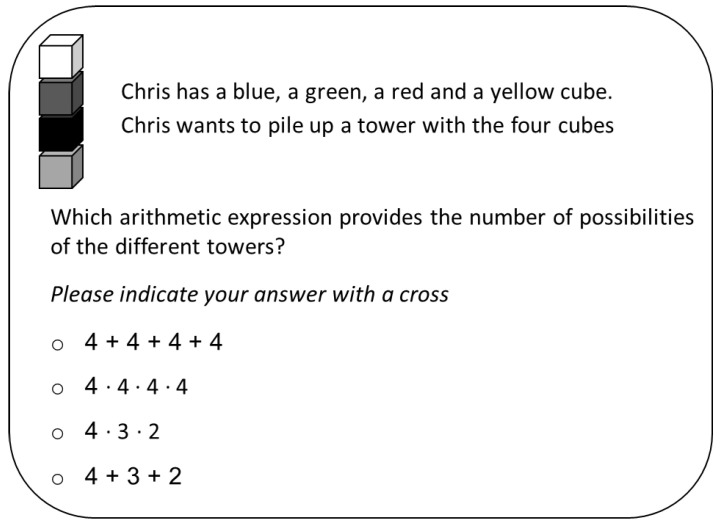
Example item from the MCK test (translated).

**Figure A2:**
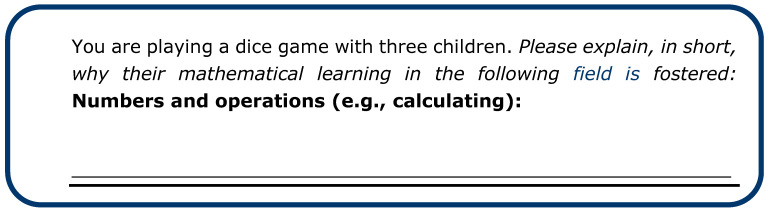
Example item from the MPCK test (translated).

**Figure A3:**
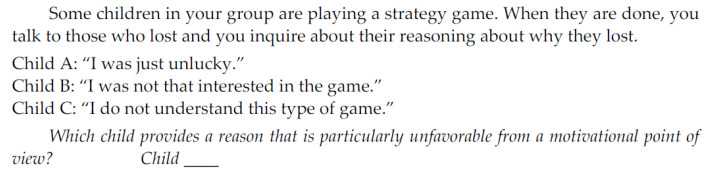
Example item from the GPK assessment (translated).

## Data Availability

The data presented in this study are available on request from the authors due to privacy restrictions.

## Note

1 Since MPCK and GPK represent conceptually different constructs, it is not appropriate to collapse these, despite very strong latent correlations. Moreover, a follow-up analysis revealed that collapsing is not supported from an empirical point of view because the fit of a uni-dimensional model was worse than a model with two distinct constructs.

## Appendix A

Item examples from the knowledge tests.
